# High expression of microRNA-183/182/96 cluster as a prognostic biomarker for breast cancer

**DOI:** 10.1038/srep24502

**Published:** 2016-04-13

**Authors:** Cailu Song, Lijuan Zhang, Jin Wang, Zhongying Huang, Xing Li, Mingqing Wu, Shuaijie Li, Hailin Tang, Xiaoming Xie

**Affiliations:** 1 Department of Breast Oncology, Sun Yat-Sen University Cancer Center, State Key Laboratory of Oncology in South China, Collaborative Innovation Center for Cancer Medicine, Guangzhou, Guangdong, China; 2 Department of Nursing, Sun Yat-Sen University Cancer Center, Guangzhou, Guangdong, China; 3 Department of Cancer Prevention Center, Sun Yat-Sen University Cancer Center, Guangzhou, Guangdong, China

## Abstract

More sensitive and effective diagnostic markers for the detection of breast cancer are urgently needed. The microRNA-183/182/96 cluster has been reported to be involved in tumorigenesis and progression in a variety of cancers, and it is a promising cancer prognostic biomarker. The goal of this study was to determine the expression levels of the miR-183/182/96 cluster in breast cancer tissues and evaluate its prognostic role in breast cancer. Real-time quantitative polymerase chain reaction analysis (qRT-PCR) was used to detect the expression levels of the miR-183/182/96 cluster in 41 breast cancer tissues and adjacent normal tissues (control tissues) and also in different mammary cell lines. *In situ* hybridization (ISH) of the miR-183/182/96 cluster on 131 tissue microarrays (TMAs) was used to statistically analyze its prognostic role. The miR-183/182/96 cluster levels were significantly higher in breast cancer tissues than in control tissues. The miR-183/182/96 cluster was also upregulated in human breast cancer cell lines. An increased miR-183/182/96 cluster level was correlated with local relapse, distant metastasis and poor clinical outcomes. Our findings improve our understanding of the expression level of the miR-183/182/96 cluster in breast cancer and clarify the role of the miR-183/182/96 cluster as a novel prognostic biomarker for breast cancer.

Breast cancer is the most common malignancy and the main cause of death among women. A total of 232, 340 new cases of invasive breast cancer and 39, 620 breast cancer deaths were expected to occur among US women in 2013^1^. From 1995 to 2006, the incidence of breast cancer gradually increased in European women in their 20s and 30 s^2^. Researchers recommended routine breast cancer screening in women younger than 50 years of age^3^. The increasing incidence of breast cancer every year causes great physiological and financial burdens for patients. The main challenge in the management of breast cancer is to identify sensitive and specific minimally invasive biomarkers that have high efficiency for screening and diagnosis and are valuable for aiding in treatment decisions.

There is increasing evidence supporting the use of microRNA (miRNA) analysis for the diagnosis and prognosis of and therapeutic decisions for breast cancer^4^,^5^,^6^. miRNAs are single-stranded RNA molecules of approximately 22 nucleotides in length. These small regulatory RNA molecules can modulate the activity of specific mRNA targets by pairing to the messenger RNAs (mRNAs) of protein-coding genes^7^. miRNAs exert posttranscriptional repression functions by binding to complementary sequences in the 3′-untranslated regions (3′-UTR) of mRNAs to promote mRNA degradation or block translation^8^. They play an important role in a wide range of physiologic and pathologic processes in animals and plants. microRNAs frequently reside in clusters that include 2–3 or more miRNA genes with pairwise chromosomal distances of up to 3000 nt in the genome^9^,^10^. Members of miRNA clusters are generally similar in sequence and transcribed in the same direction. They are highly conserved and usually function synergistically^11^.

The miR-183/182/96 cluster is composed of 3 miRNA genes located in a 4-kb region of mouse chromosome 6qA3^12^ and located in a 5-kb region of human chromosome 7q32.2. Several studies have confirmed that members of the miR-183/182/96 cluster are abnormally expressed in many tumors and are closely related to human cancers. Each member of the miR-183/182/96 cluster can function as an oncogene or anti-oncogene, depending on the cancer type, location and stage^13^. miRNA-183 has been reported to promote migration and invasion in osteosarcoma^14^ and to be correlated with shorter overall survival in prostate cancer^15^. miRNA-182 has been shown to promote aggression in glioma^16^ and migration and survival in melanoma^17^. miRNA-96 was shown to increase proliferation and colony formation in hepatocellular carcinoma^18^. The members of the human miR-183/182/96 cluster have been reported to be biomarkers for prostate cancer^19^, bladder cancer^20^ and urothelial carcinoma^21^. Overall, the role of the miR-183/182/96 cluster in cancer is complex. Increased expression of this cluster was implicated in glioma carcinogenesis^22^. In most types of breast cancers, overexpression of the miR-183/182/96 cluster has been reported to increase cell proliferation and migration. Thus, the members of this cluster serve as oncogenes in breast cancer^13^.

Although it is well known that the expression level of the miR-183/182/96 cluster is increased in several tumor types, its prognostic role in breast cancer is still unclear. In this study, we investigated the expression levels of the miR-183/182/96 cluster in breast cancer tissues and adjacent normal tissues. The expression levels of the miR-183/182/96 cluster were also studied in multiple mammary cell lines. Then, we performed a preliminarily evaluation of its prognostic role by statistically analyzing tissue microarray results. Furthermore, we evaluated the OS and DFS of breast cancer patients with high and low expression of the miR-183/182/96 cluster to further judge its prognostic role for breast cancer.

## Results

### miR-183/182/96 cluster was upregulated in breast cancer cell lines and clinical specimens

**Figure 1:**
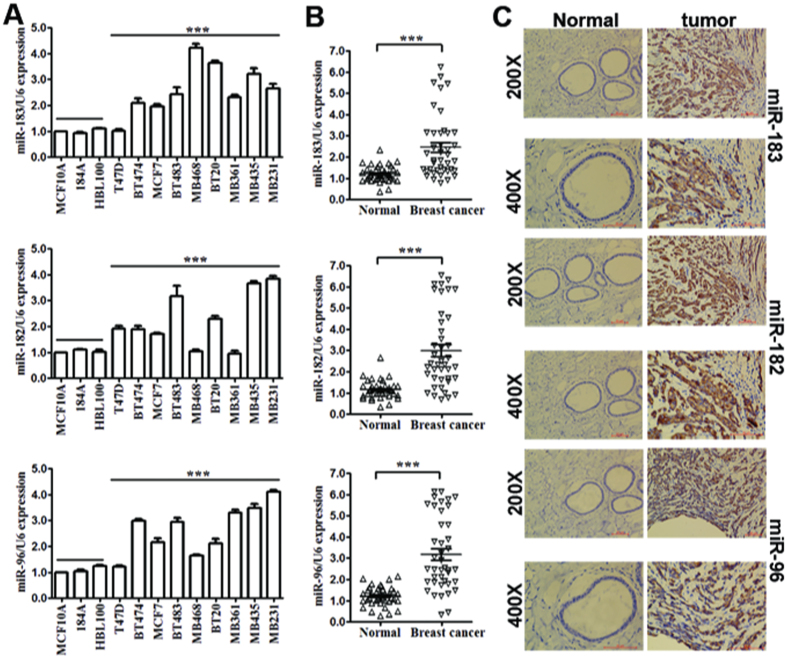
miR-183/182/96 cluster was upregulated in breast cancer clinical specimens and cell lines. (**A**) Expression levels of miR-183/182/96 in 12 different mammary cell lines detected by qRT-PCR analysis. (**B**) Expression levels of miR-183/182/96 in 41 pairs of breast cancer tissues and adjacent normal tissues (control tissues) detected by qRT-PCR analysis. All data are shown as the mean ± SE. ***P < 0.01. (**C**) Representative ISH images of miR-183/182/96 under a microscope (200× and 400×).

qRT-PCR analysis was used to detect the expression of miR-183/182/96 in 12 different mammary cell lines, including human breast cancer cell lines (MDA-MB-435, MDA-MB-361, MDA-MB-231, BT-483, BT-474, BT-20, MCF-7, MDA-MB-468 and T-47D) and human mammary epithelial (HME) cell lines (BHL-100, 184A and MCF-10A). We found that miR-183/182/96 was upregulated in human breast cancer cell lines compared to in HME cell lines (Fig. 1A).

Then, we detected the expression of miR-183/182/96 in 41 pairs of breast cancer tissues and adjacent normal tissues (control tissues). Among 41 breast cancer patients, approximately 82.9% (p < 0.01, 34 of 41 patients), 82.9% (p < 0.01, 34 of 41 patients) and 87.8% (p < 0.01, 36 of 41 patients) of tumor samples showed significant increases in miR-183, miR-182 and miR-96 levels, respectively (Fig. 1B). These results indicated that upregulation of miR-183/182/96 was a frequent event in breast cancer clinical specimens and could be related to breast cancer carcinogenesis.

Subsequently, we examined miR-183/182/96 expression by ISH. We observed that both nuclear and cytoplasmic staining of miR-183/182/96 was more frequently observed in tumor cells than in normal mammary epithelial cells. Representative ISH images of miR-183/182/96 under a microscope are shown (Fig. 1C, 200×and 400×). Upregulation of miR-183/182/96 was a frequent event in the breast cancer clinical specimens examined and could be related to breast cancer carcinogenesis.

### Increased miR-183/182/96 level was correlated with TNM stage, local relapse and distant metastasis

We researched the potential clinicopathological implications of altered miR-183/182/96 expression. The tissue microarrays (TMAs) of 131 breast cancer samples were used for *In Situ* Hybridization (ISH) and Immunohistochemistry (IHC) analysis. These clinical samples were divided into low or high expression groups with a miR-183/182/96 expression cutoff score of 2. Chi-square tests were used to analyze the relationship between clinical pathological parameters and altered miR-183, miR-182, and miR-96 levels (Table 1). Among 131 breast cancer patients, we found that miR-183, miR-182 and miR-96 levels were positively correlated with both TNM stage (*P* = 0.012, 0.018 and 0.020, respectively) and distant metastasis (*P* = 0.000, 0.001 and 0.033, respectively) (Table 1) but not with tumor size; LNMET (lymph node metastasis); age; menopause; or ER, PR or HER2 status. miR-183 and miR-182 were positively correlated with local relapse (*P* = 0.025 and 0.028, respectively). Only miR-96 was positively correlated with P53 status (*P* = 0.014). These results demonstrate that miR-183/182/96 might play a crucial role in the occurrence and progression of breast cancer.

**Figure 2:**
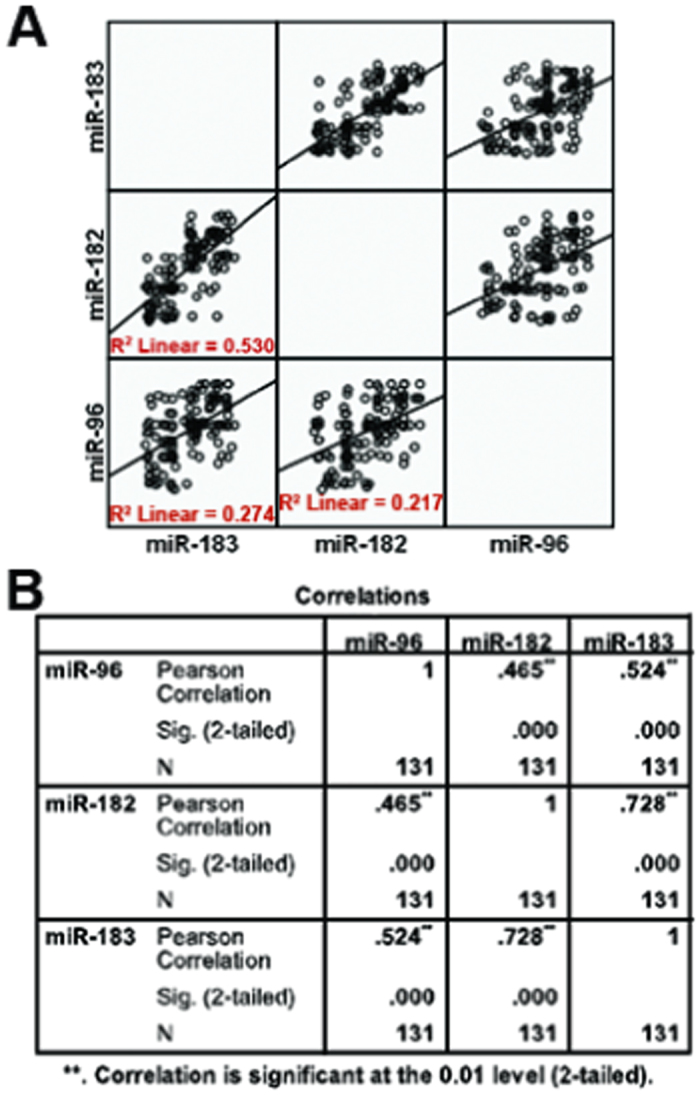
Increased miR-183/182/96 cluster level was correlated with clinical stage, local relapse and distant metastasis. (**A**) Pearson correction analysis results in the form of image. Pearson correction analysis was used to analyze the correlations between miR-183, miR-182 and miR-96 expression levels. The correlation coefficient between miR-183 and miR-182 was the highest. (**B**) Pearson correction analysis results in the form of chart. **Correlation is significant at the 0.01 level (2-tailed).

Then, we explored the correlation between miR-183, miR-182 and miR-96 expression using Pearson correction analysis. The correlation coefficient between miR-183 and miR-182 was 0.728 (linear R^2^ = 0.530), that between miR-183 and miR-96 was 0.524 (linear R^2^ = 0.274), and that between miR-182 and miR-96 was 0.465 (linear R^2^ = 0.217), respectively; indicating that the expression levels of miR-182 and miR-96 or miR-183 and miR-182 were more significantly related than those of miR-183 and miR-96 (2-tailed, Fig. 2A,B).

### Increased miR-183/182/96 level was correlated with poor clinical outcomes

**Figure 3:**
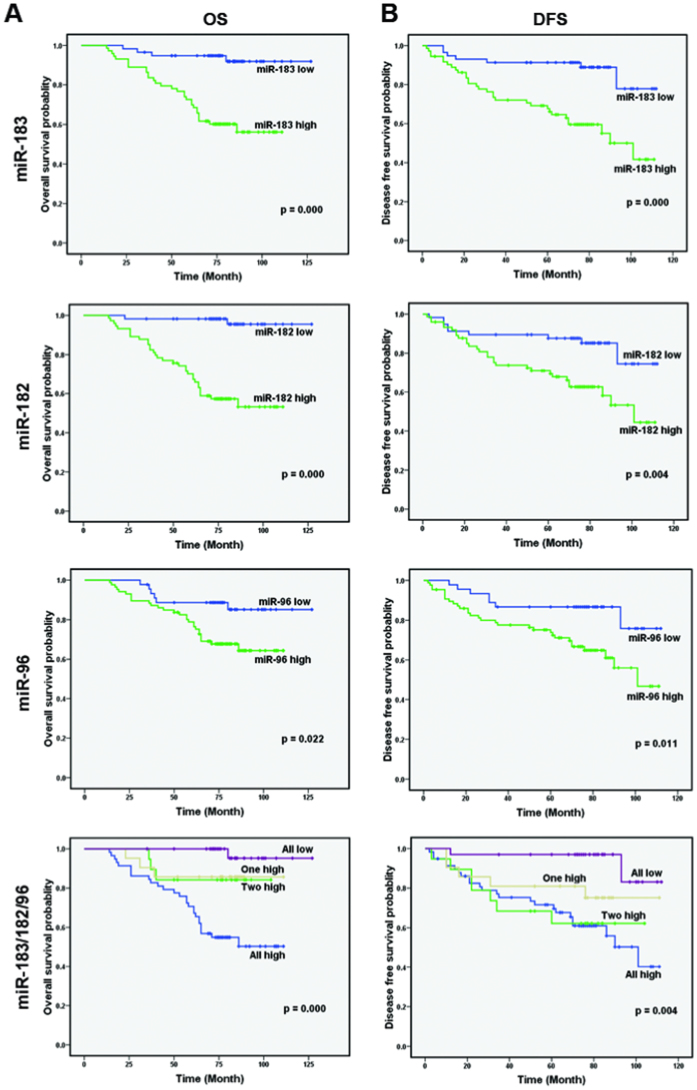
Increased miR-183/182/96 level was correlated with poor prognosis. (**A**) OS curves for 131 patients with low or high miR-183, miR-182, miR-96 and miR-183/182/96 cluster expression. Kaplan-Meier and log-rank analyses were used. High levels of the miR-183/182/96 cluster were markedly correlated with shorter overall survival. (**B**) DFS curves for 131 patients with low or high miR-183, miR-182, miR-96 and miR-183/182/96 cluster expression. A high level of the miR-183/182/96 cluster was markedly correlated with shorter disease-free survival. All data are shown as the mean ± SE ***P < 0.01.

The results above revealed that miR-183/182/96 level was higher in breast cancer and was positively correlated with some clinical pathological parameters, indicating that miR-183/182/96 may be vital for the pathogenesis of breast cancer. To further explore the prognostic significance of miR-183/182/96, we used Kaplan-Meier survival analysis to generate patient overall survival (OS) and disease-free survival (DFS) curves. The results demonstrated that patients with higher miR-183, miR-182 and miR-96 expression levels had shorter mean OS and DFS than patients with lower expression levels (*P* = 0.000, 0.000 and 0.022 for OS, respectively and *P* = 0.000, 0.004 and 0.011 for DFS, respectively, Fig. 3A,B). Further more, when the expression levels of all three members of the miR-183/182/96 cluster were high, OS and DFS were much shorter than when zero, one, or two members of the miR-183/182/96 cluster were highly expressed (all *P* for OS = 0.000 and all *P* for DFS = 0.004). These results demonstrated that miR-183/182/96 expression was significantly associated with patient OS and DFS. The miR-183/182/96 cluster is a potential prognostic biomarker for breast cancer.

## Discussion

As important regulatory molecules, miRNAs have been repeatedly confirmed to be involved in disease pathogenesis^23^. miRNAs are highly conserved and stable in cells and tissues. Also, they can be easily detected and extracted^24^. Thus, miRNAs are commonly chosen as potential biomarkers for the detection and monitoring of the diagnosis and prognosis of and therapeutic decisions for different cancers^4^,^25^. Members of miRNA clusters function synergistically. Currently, little is known regarding the expression of the miR-183/182/96 cluster in most cancers.

In this study, we found that the expression level of the miR-183/182/96 cluster was upregulated in breast cancer tissues and cell lines in comparison to the corresponding adjacent normal tissues and normal mammary cell lines, respectively, using qRT-PCR analysis. These results are consistent with a previous report that the miR-183/96/182 cluster is overexpressed in prostate tissue^26^.

miR-183, miR-182 and miR-96 have been proposed as oncogenes in glioma^22^ and medulloblastoma^27^. The miR-183/182/96 cluster regulates the proliferation of colon cancer cells^28^. Each member of the miR-183/182/96 cluster has been reported to promote migration, invasion or metastasis in different carcinomas^14^,^16^,^18^. Thus, it is highly likely that the miR-183/182/96 cluster has a close relationship with various clinical pathology indices. We evaluated the prognostic role of miR-183/182/96 using Pearson correction analysis. An increased level of the miR-183/182/96 cluster was correlated with both clinical stage and distant metastasis. We also found that the expression levels of miR-183 and miR-182 were more significantly related than those of miR-182 and miR-96 or miR-183 and miR-96. This finding coincided with the results that local relapse was positively correlated with miR-183 and miR-182, but not with miR-96.

Members of the miR-183/182/96 cluster have been reported as blood biomarkers of retinal toxicity^29^. The expression levels of the members of the miR-183/182/96 cluster were shown to be associated with significantly lower rates of event-free and overall survival in medulloblastoma^27^. Here, we used Kaplan-Meier survival methods to analyze patient overall survival (OS) and disease-free survival (DFS). We found that patients with higher miR-183/182/96 expression had shorter mean OS and DFS than patients with lower miR-183/182/96 expression. OS and DFS were shortest when the levels of all three members of the miR-183/182/96 cluster were high. Thus, the miR-183/182/96 cluster has potential for use in the diagnosis and prognosis of and therapeutic decisions for breast cancer.

The expression level of the miR-183/182/96 cluster is high in breast cancer tissues and cell lines. Poor prognosis and clinical pathological parameters of breast cancer patients were closely related to high expression of the miR-183/182/96 cluster. The miR-183/182/96 cluster may be used in the future as a novel breast cancer biomarker to guide tumor diagnosis, treatment and prognosis predication. Our findings improve our understanding of the expression level of the miR-183/182/96 cluster in breast cancer. The miR-183/182/96 cluster has potential for use as a novel prognostic biomarker for breast cancer.

## Methods

### Clinical samples

All human breast cancer tumor samples were obtained from randomly selected cancer patients at the Sun Yat-Sen University Cancer Center (SYSUCC), Guangzhou, China. The diagnoses of breast cancer were pathologically confirmed in all cases. The patients had not received any prior chemotherapy. Tissues samples were collected intraoperatively. Written informed consent was obtained from all eligible patients who participated in the study before or after surgery, and all protocols were reviewed and approved by the Joint Ethics Committee of the Sun Yat-Sen University Cancer Center and used according to the ethical standards as formulated in the Helsinki Declaration.

All the clinical data of patients such as ER/PR/HER2 status, age, tumor size, histologic type, lymph node status, local or distant metastatic relapse were available and reviewed. Histologic type was determined on TNM staging system which was classified according to the WHO classification and tumor stage (American Joint Committee on Cancer classification). The patients were grouped according to their clinical features listed in Table 1.

### Cell lines and culture

Human breast cancer cell lines (MDA-MB-435, MDA-MB-361, MDA-MB-231, BT-483, BT-474, BT-20, MCF-7, MDA-MB-468 and T-47D) and human mammary epithelial (HME) cell lines (BHL-100, 184A and MCF-10A) were obtained from the American Type Culture Collection (Manassas, VA, USA). All cells were passaged in our laboratory for less than six months after resuscitation of frozen aliquots. The cells were cultured in Dulbecco’s modified Eagle’s medium (DMEM, Invitrogen, CA, USA) supplemented with 10% fetal bovine serum (FBS, Gibco, Cappinas, Brazil) in a humidified incubator at 37°C containing 5% CO_2_.

### *In situ* hybridization analysis

miR-183/182/96 miRCURYTM LNA custom detection probes (Exiqon, Vedbaek, Denmark) were used for *in situ* hybridization (ISH). Hybridization, washing, and scanning were performed according to the manuals and protocols provided by the Exiqon Life Science Department, and staining assessments were performed by two independent pathologists. Staining intensity was scored as 0 (negative), 1+ (weak), 2+ (medium) or 3+ (strong). Low expression was defined as an intensity of 0, 1, 2, or 3 with <10% stained cells or by an intensity of 0 or 1 with <50% stained cells. High expression was defined as an intensity of 2 or 3 with >10% stained cells or by an intensity of 1, 2, or 3 with >50% stained cells.

### miRNA isolation from cell lines and tissues

Tissue samples were homogenized in a denaturing lysis solution. Total RNA was extracted from the lysed tissue using TRIzol reagent (Invitrogen Life Technologies, Carlsbad, CA, USA). Then, miRNA was separated from 30–50 mg of total RNA using the Ambion miRNA Isolation Kit (Ambion, Carlsbad, CA, USA). Tissues samples were stored at 80 °C. miRNAs were extracted from cell lines and tissues using the mirVana™ miRNA isolation kit (Ambion) according to the manufacturer’s instructions.

### Real-time quantitative PCR analysis

Expression levels of miR-183/182/96 were detected in both breast cancer tissues and cell lines. 41 pairs of breast cancer tissues and adjacent normal tissues (control tissues) from breast cancer patients were obtained. Human breast cancer cell lines (MDA-MB-435, MDA-MB-361, MDA-MB-231, BT-483, BT-474, BT-20, MCF-7, MDA-MB-468 and T-47D) and human mammary epithelial (HME) cell lines (BHL-100, 184A and MCF-10A) were also included. Total RNAs were extracted from the cells and tissues using TRIzol reagent (Invitrogen, Carlsbad, CA, USA). The RT-PCR reactions were performed using a qSYBR Green-containing PCR kit (Invitrogen, Carlsbad, CA, USA). The expression of miR-183/182/96 was quantified by measuring cycle threshold (Ct) values and was normalized to U6-snRNA or β-actin (endogenous control for miRNA detection) using the 2^−ΔΔdt^ method.

### Statistical analysis

The qRT-PCR assays were performed in triplicate. Each experiment was repeated several times, and the results are presented as the mean ± SE. The statistical analysis was performed using Student’s *t*-test. Using the Kaplan-Meier method, overall survival (OS) and disease-free survival (DFS) curves were plotted. The significance level was set at *p* < 0.05. All statistical analyses were accomplished using SPSS 17.0 software.

## Additional Information

**How to cite this article**: Song, C. *et al.* High expression of microRNA-183/182/96 cluster as a prognostic biomarker for breast cancer. *Sci. Rep.* **6**, 24502; doi: 10.1038/srep24502 (2016).

## Figures and Tables

**Table 1:** Clinicopathological variables and miR-183/182/96 expression in 131 breast cancer patients.

Characteristics	Total (n = 131)	miR-183 High/Low	P value	miR-182 High/Low	P value	miR-96 High/Low	P value
		(73/58)		(74/57)		(86/45)	
OS			0.000*		0.000*		0.022*
Present	97	43/54		42/55		58/39	
Absent	34	30/4		32/2		28/6	
DFS			0.000*		0.004*		0.011*
Present	93	42/51		45/48		55/38	
Absent	38	31/7		29/9		31/7	
Age (years)			0.439		0.084		0.410
<50	81	43/38		41/40		51/30	
>=50	50	30/20		33/17		35/15	
Menopause			0.842		0.071		0.822
Yes	60	34/26		39/21		40/20	
No	71	39/32		35/36		46/25	
Tumor size (cm)			0.809		0.969		0.129
=<2	37	20/17		21/16		28/9	
>2	94	53/41		53/41		58/36	
LNMET			0.745		0.592		0.089
Yes	77	42/35		42/35		46/31	
No	54	31/23		32/22		40/14	
TNM stage			0.012*		0.018*		0.020*
I–II	72	33/39		34/38		41/31	
III–IV	59	40/19		40/19		45/14	
Local relapse			0.025*		0.028*		0.849
Yes	8	8/0		8/0		6/2	
No	123	65/58		66/57		80/43	
Distant metastasis			0.000*		0.001*		0.033*
Yes	32	27/5		26/6		26/6	
No	99	46/53		48/51		60/39	
ER status			0.635		0.180		0.752
Positive	49	26/23		24/25		33/16	
Negative	82	47/35		50/32		53/29	
PR status			0.725		0.115		0.959
Positive	52	28/24		25/27		34/18	
Negative	79	45/34		49/30		52/27	
HER-2 status			0.553		0.501		0.784
Positive	22	11/11		11/11		15/7	
Negative	109	62/47		63/46		71/38	
P53 status			0.745		0.590		0.014*
Positive	77	42/35		45/32		44/33	
Negative	54	31/23		29/25		42/12	

^*^Means statistically significant (P < 0.05).
